# Longitudinal Growth Differentiation Factor 15 (GDF15) and Long-term Intraocular Pressure Fluctuation in Glaucoma: A Pilot Study

**DOI:** 10.18502/jovr.v16i1.8245

**Published:** 2021-01-20

**Authors:** Jonathan B. Lin, Arsham Sheybani, Andrea Santeford, Rajendra S. Apte

**Affiliations:** ^1^Departments of Ophthalmology and Vision Science, Washington University, USA; ^2^Departments of Developmental Biology, Washington University, USA; ^3^Departments of Medicine, Washington University, USA

**Keywords:** GDF15, Glaucoma, Neurodegeneration, Molecular Markers

## Abstract

**Purpose:**

Growth Differentiation Factor 15 (GDF15) was previously identified as a molecular marker of retinal ganglion cell stress in rodent models of glaucoma and was elevated in the aqueous humor (AH) of patients with primary open-angle glaucoma as a possible risk factor for glaucoma progression. The purpose of this study was to determine whether changes in the AH GDF15 levels were associated with intraocular pressure (IOP) changes in eyes undergoing glaucoma surgery.

**Methods:**

Here, we performed a prospective, longitudinal pilot study in nine patients to determine whether changes in AH GDF15 levels from surgery to post-surgery follow-up were associated with IOP fluctuation. An initial AH sample was taken from the peripheral corneal paracentesis during planned glaucoma surgery, and a second sample was taken during an outpatient follow-up visit, approximately six months later.

**Results:**

There was a statistically significant correlation between GDF15 fold change and IOP standard deviation (*r* = 0.87, *P* = 0.003), IOP range (*r* = 0.87, *P* = 0.003), and maximum IOP (*r* = 0.86, *P* = 0.003). There was no correlation between the GDF15 fold change and baseline IOP (*r* = 0.50, *P* = 0.17), final IOP (*r* = 0.038, *P* = 0.92), or mean IOP (*r* = 0.40, *P* = 0.28).

**Conclusion:**

Our findings in this pilot study suggest that longitudinal changes in AH GDF15 may be associated with IOP fluctuation during the postoperative period. Further studies are necessary to corroborate these findings in a larger patient population and to explore the possibility that AH GDF15 may be used not only to improve treatment algorithms but also as a surrogate endpoint in clinical trials.

##  INTRODUCTION

Glaucoma is a neurodegenerative disease characterized by progressive death of retinal ganglion cells (RGCs). Although it is currently the second leading cause of blindness worldwide,^[1]^ the molecular pathogenesis of RGC death remains elusive. Therefore, interventions are currently centered on lowering intraocular pressure (IOP), a risk factor for disease progression.^[2]^ Most clinical decision-making is based upon measuring IOP and surrogates of glaucomatous neurodegeneration, such as Humphrey visual field, cup-to-disc ratio, and nerve fiber layer thickness. Unfortunately, these surrogate metrics are imprecise in their ability to quantify disease severity and, in some cases, are subjective and unreliable. Therefore, there is a clinical need for molecular markers that measure RGC health and stress prior to cell death to guide optimal medical and surgical management of glaucoma patients.

It was previously reported that Growth Differentiation Factor 15 (GDF15), a member of the Transforming Growth Factor beta (TGF-β) superfamily, is a molecular marker of RGC stress in rodent models of glaucoma.^[3]^ Validation studies in well-characterized human patients showed that GDF15 levels not only were elevated in the aqueous humor (AH) of primary open-angle glaucoma (POAG) patients compared to control patients without glaucoma but also increased stepwise with increasing visual field loss by Hodapp-Parrish-Anderson staging.^[3]^ However, because of the cross-sectional study design, they were unable to determine whether changes in AH GDF15 levels were associated with IOP changes, which have been reported as possible risk factors for progression. To explore this possibility, we performed a prospective, longitudinal pilot study to determine whether changes in AH GDF15 levels over a follow-up period of approximately six months are associated with IOP changes in eyes undergoing glaucoma surgery.

##  METHODS

We recruited nine participants from one large academic institution. All patients gave written informed consent. This study was approved by the Institutional Review Board (IRB) of the Human Research Protection Office (HRPO) of the local Ethics Committee. All procedures adhered to the tenets of the Declaration of Helsinki. Patients were included if they had any form of glaucoma, including POAG or secondary glaucoma, and were determined to be candidates for MoltenoⓇ glaucoma implant (Molteno Ophthalmic Limited, Dunedin, New Zealand) or AhmedⓇ glaucoma valve (New World Medical, Rancho Cucamonga, CA) surgery. Eyes were excluded if there was active inflammatory eye disease, any retinopathy, or any optic nerve degeneration from non-glaucomatous causes. To determine appropriate sample size, we performed a power analysis using G*Power 3.1.9.2.^[15]^ Estimating an effect size of *r* = 0.75 based on previous data, we calculated a sample size of *N* = 9 to achieve 80% power at a two-tailed alpha of 0.05.

Two AH samples were obtained from each patient. The first AH sample was obtained in the operating room during planned glaucoma surgery. Briefly, a blunt cannula attached to a tuberculin syringe was inserted into the initial peripheral corneal paracentesis and used to remove 50–100 µl of AH. The second AH sample was obtained during a clinic visit, approximately six months after the initial surgery. Briefly, using sterile technique, a needle on a syringe was used to enter the anterior chamber temporally, anterior to the limbus, to gently aspirate AH, with care taken to not deform the anterior chamber. In both cases, AH samples were immediately placed on dry ice and then stored at –80ºC until further analysis. We measured GDF15 levels of all AH samples at the same time using the commercially available human GDF15 Quantikine enzyme-linked immunosorbent assay (ELISA) kit (R&D Systems), as described previously.^[3]^ The individual performing GDF15 measurements (XXX) was masked to demographic and clinical information to minimize bias.

Demographic information, clinical information, and IOP measurements were obtained by retrospective chart review. All IOP values were measured by Goldmann Applanation Tonometry, performed by ophthalmologists or optometrists who were masked to the study data. Participants had IOP measurements taken as a part of routine clinical care at postoperative day 1, postoperative week 1, postoperative month 1, and additional follow-up visits as clinically indicated. The primary variables of interest were measures of IOP fluctuation that have been previously reported as risk factors for glaucoma progression, such as IOP standard deviation, IOP range, and maximum IOP. We also analyzed baseline IOP (measured at the clinic visit prior to glaucoma surgery), final IOP (measured at the same clinic visit during which the second AH sample was collected), and mean IOP over the follow-up period.

We performed statistical analysis and data visualization with R Version 3.6.2 and RStudio Version 1.2.5003. To compare means between two groups, we used the Mann–Whitney U test due to small sample size. To compare pre- and post-surgery number of medications, we used the Wilcoxon signed-rank test. To determine associations between continuous variables, we calculated Pearson product-moment correlation coefficients. Because of relatively small sample sizes, we also calculated Kendall rank correlation coefficients to confirm our results. We considered *P* < 0.05 to be statistically significant.

##  RESULTS

Demographic and clinical characteristics of the participants are shown in Table 1. There were four male and five female participants. The mean age was 71.0 years (standard deviation: 9.6 years). Eight patients had POAG, while one patient had glaucoma secondary to presumed herpes simplex uveitis/trabeculitis, which had been inactive for greater than three months. Three patients underwent placement of a MoltenoⓇ glaucoma implant; six patients underwent placement of an AhmedⓇ glaucoma valve. Three patients underwent surgery in their left eye; six in the right eye. The mean follow-up duration was 183.4 days (standard deviation: 28.0 days, minimum: 131 days, maximum: 215 days). Patients were on significantly fewer classes of medications after surgery compared to before surgery (*P* = 0.013).

**Table 1:** Demographic and clinical characteristics of study participants


**Characteristic**	**Value**
Age, Mean ± SD${}^{a}$	71.0 ± 9.6
Sex, *N* ${}^{b}$ (%)	
Male	4 (44.4)
Female	5 (55.6)
Type of Glaucoma, *N* (%)	
Primary open-angle glaucoma	8 (88.9)
Glaucoma secondary to inflammation	1 (11.1)
Type of Procedure, *N* (%)	
MoltenoⓇ glaucoma implant	3 (33.3)
AhmedⓇ glaucoma valve	6 (66.7)
Study Eye, *N* (%)	
OS	3 (33.3)
OD	6 (66.7)
Pre-surgery Medication Classes, Median (Range)	4 (2 – 4)
Post-surgery Medication Classes, Median (Range)	2 (0 – 3)${}^{c}$
${}^{a}$SD: standard deviation;${}^{b}$N: number of participants; ${}^{c}$There is a significant difference between pre- and post-surgery number of medication classes by the Wilcoxon signed rank test: *P* = 0.013	

**Figure 1:**
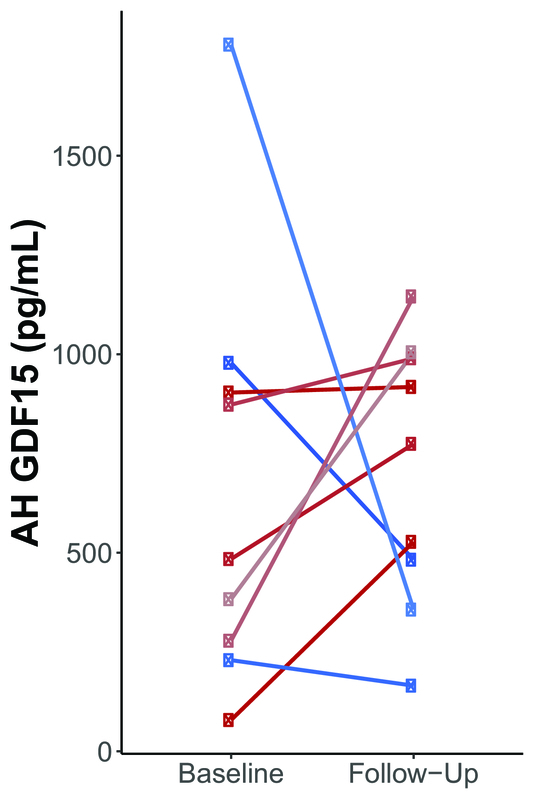
Of the nine participants, six had increased aqueous humor (AH) Growth Differentiation Factor 15 (GDF15) from baseline to follow-up at approximately six months (shades of red), while three had decreased AH GDF15 (shades of blue). Circles denote patients who received the AhmedⓇ glaucoma valve; squares denote patients who received the MoltenoⓇ glaucoma implant.

**Figure 2:**
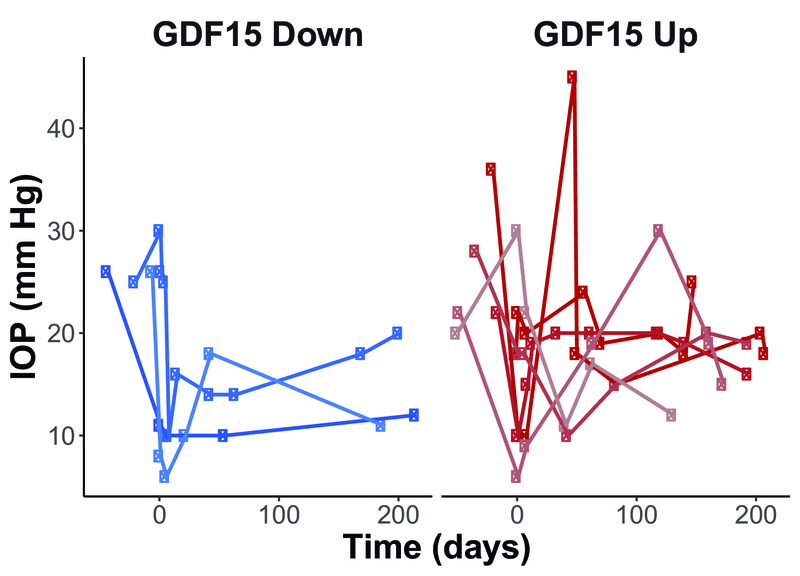
Serial intraocular pressure measurements by Goldmann Applanation Tonometry for participants whose aqueous humor (AH) Growth Differentiation Factor 15 (GDF15) increased (“GDF15 Up”; *N* = 6; shades of red) and those whose AH GDF15 decreased (“GDF15 Down”; *N* = 3; shades of blue). Day 0 was set as the day of glaucoma surgery when the initial AH sample was collected. Circles denote patients who received the AhmedⓇ glaucoma valve; squares denote patients who received the MoltenoⓇ glaucoma implant.

**Figure 3:**
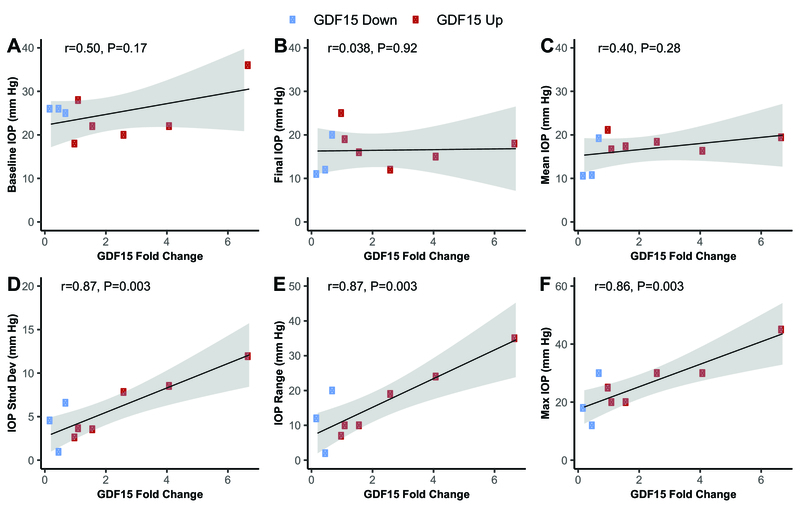
Aqueous humor Growth Differentiation Factor 15 (GDF15) fold change from baseline to six-month follow-up was not correlated with baseline intraocular pressure (IOP; A), final IOP (B), or mean IOP (C). In contrast, there was a strong correlation between GDF15 fold change and IOP standard deviation (stnd dev; D), IOP range (E), and maximum (max) IOP (F). *r* = Pearson correlation coefficients. Similar significance levels were found with non-parametric Kendall rank correlation coefficients. Shaded regions indicate 95% confidence interval bands. Circles denote patients who received the AhmedⓇ glaucoma valve; squares denote patients who received the MoltenoⓇ glaucoma implant.

There was no significant correlation between baseline AH GDF15 and baseline IOP, mean IOP, or final IOP (*P* > 0.05). Similarly, there was no significant correlation between follow-up AH GDF15 and baseline IOP, mean IOP, or final IOP (*P* > 0.05). Of the nine participants, six had increased AH GDF15 levels over the follow-up interval, while three had decreased AH GDF15 levels at follow-up approximately six months later [Figure 1]. All participants had between four to nine IOP measurements during the follow-up period [Figure 2]. When dichotomizing participants into those who had increased (“GDF15 Up”; *N* = 6) versus decreased (“GDF15 Down”; *N* = 3) AH GDF15 levels, there were no statistically significant differences in the baseline IOP, final IOP, mean IOP, IOP standard deviation, IOP range, or maximum IOP (*P* > 0.05 by Mann–Whitney U tests). GDF15 fold change from baseline to follow-up was not correlated with baseline IOP, final IOP, or mean IOP [Figures 3A–C]. In contrast, GDF15 fold change was strongly correlated with IOP standard deviation, IOP range, and maximum IOP [Figures 3D–F] with statistical significance achieved with both parametric and non-parametric tests.

##  DISCUSSION

In this prospective, observational pilot study, we analyzed whether changes in AH GDF15 levels from baseline to follow-up at approximately six months were associated with IOP fluctuation. Our findings suggest that AH GDF15 fold change is indeed associated with IOP fluctuation. IOP fluctuation that occurs over months to years has been reported as a risk factor for visual field progression in glaucoma in the Advanced Glaucoma Intervention Study (AGIS),^[4,5]^ the Collaborative Initial Glaucoma Treatment Study (CIGTS),^[6]^ and the Japanese Archive of Multicentral Databases in Glaucoma (JAMDIG).^[7]^ Thus, our findings suggest that increases in AH GDF15 measurements may be associated with increased risk of glaucoma progression. Although not all studies have corroborated IOP fluctuation as a risk factor for glaucoma progression, Kim and Caprioli previously hypothesized that this discrepancy may be due to higher mean IOP in some study populations that could potentially mask the effect of IOP fluctuation.^[8]^

Given this possible role of IOP fluctuation in glaucoma progression, especially for patients who show progression despite having IOPs near goal, the ability to use a molecular marker such as GDF15 as a marker of long-term IOP fluctuation is highly desirable. Routine IOP measurements to assess for fluctuation is time-consuming given the need for repeated clinic visits and is rarely performed outside of clinical trials due to demands not only for the clinician but also for patients and their families. Home tonometer devices such as the IcareⓇ HOME tonometer (Icare USA, Raleigh, NC) are available but have uncertain reliability. Additionally, devices such as the TriggerfishⓇ Contact Lens Sensor (SENSIMED, Lausanne, Switzerland) can measure changes in ocular dimensions thought to be related to IOP but is typically used for only a 24-hr period and is still experimental, as studies investigating their correlation with IOP measurements obtained through other validated methods have yielded mixed results.^[9]^

Although glaucoma is one of the leading causes of blindness worldwide, identifying reliable molecular markers has been challenging.^[10]^ This lack of molecular markers has led to reliance on surrogate markers of glaucomatous neurodegeneration for clinical decision-making, even though these surrogate markers are imprecise and sometimes unreliable. Additionally, there is a great need for novel molecular markers of RGC health that can be used as reliable surrogate endpoints for clinical trials.^[11]^ Although further validation is necessary to demonstrate a direct link to glaucoma progression, we propose that AH GDF15 may be a molecular marker of long-term IOP fluctuation that may be used in future therapeutic trials.

One limitation of the present study is the relatively small sample size. Although we achieved the necessary sample size for adequate statistical power, our small sample size does not permit us to control for possible covariates, such as age and gender. Another limitation of the study is the heterogeneity of glaucoma subtype and the type of surgery that these patients underwent. We cannot rule out the possibility that differences in the underlying disease pathophysiology or underlying differences of the post-operative IOP profiles of the AhmedⓇ glaucoma valve versus the MoltenoⓇ glaucoma implant may have influenced our findings. Future longitudinal studies in larger populations are necessary to address these limitations and may also incorporate functional testing to directly measure glaucoma progression.

One strength of our study is that we have a well-characterized patient population for whom we have longitudinal GDF15 measurements. This within-subjects design allowed us to account for inter-individual variability since GDF15 has shown to be elevated in other contexts, such as neurodegenerative and cardiovascular disease.^[12,13]^

Although many groups have explored AH biomarkers for numerous ocular diseases,^[14]^ many of these studies have analyzed samples obtained during cataract or glaucoma surgery. It is important to note that it is possible to collect AH in the outpatient setting. This procedure has minimal risks when performed by an experienced practitioner and was well tolerated by participants in this study. Although it is somewhat invasive, we propose that the ability to quantitatively assess RGC health may outweigh any risks associated with such as a procedure.

##  Resource Availability

The data analyzed in this study are available from the corresponding author on reasonable request.

##  Financial Support and Sponsorship

None.

##  Conflicts of Interest

There are no conflicts of interest.
